# Hantavirus–*Leptospira* coinfections in small mammals from central Germany

**DOI:** 10.1017/S0950268821000443

**Published:** 2021-02-22

**Authors:** K. Jeske, J. Jacob, S. Drewes, M. Pfeffer, G. Heckel, R. G. Ulrich, C. Imholt

**Affiliations:** 1 Institute of Novel and Emerging Infectious Diseases, Friedrich-Loeffler-Institut, Federal Research Institute for Animal Health, Südufer 10, 17493 Greifswald-Insel Riems, Germany; 2 Institute of Diagnostic Virology, Friedrich-Loeffler-Institut, Federal Research Institute for Animal Health, Südufer 10, 17493 Greifswald-Insel Riems, Germany; 3 Vertebrate Research, Institute for Plant Protection in Horticulture and Forests, Julius Kühn-Institute, Toppheideweg 88, 48161 Münster, Germany; 4 Institute of Animal Hygiene and Veterinary Public Health, University of Leipzig, An den Tierkliniken 1, 04103 Leipzig, Germany; 5 Institute of Ecology and Evolution, University of Bern, Baltzerstrasse 6, 3012 Bern, Switzerland

**Keywords:** Coinfection, common vole, *Leptospira* spp., Puumala orthohantavirus, Tula orthohantavirus

## Abstract

European orthohantaviruses (Puumala orthohantavirus (PUUV); Dobrava-Belgrade orthohantavirus (DOBV), genotype Kurkino; Tula orthohantavirus (TULV)), and *Leptospira* spp. are small mammal-associated zoonotic pathogens that cause diseases with potentially similar symptoms in humans. We investigated the frequency of *Leptospira* spp. and hantavirus single and double infections in small mammals from 22 sites in Thuringia, central Germany, during 2017. TULV infections were detected at 18 of 22 sites (mean prevalence 13.8%, 93/674). PUUV infections were detected at four of 22 sites (mean prevalence 1.5%, 7/471), and respective PUUV sequences formed a novel phylogenetic clade, but DOBV infections were not detected at all. *Leptospira* infections were detected at 21 of 22 sites with the highest overall prevalence in field voles (*Microtus agrestis*) with 54.5% (6/11) and common voles (*Microtus arvalis*) with 30.3% (205/676). *Leptospira*–hantavirus coinfections were found in 6.6% (44/671) of common voles but only in two of 395 bank voles. TULV and *Leptospira* coinfection probability in common voles was driven by individual (age) and population-level factors. Coinfections seemed to be particularly associated with sites where *Leptospira* spp. prevalence exceeded 35%. Future investigations should evaluate public health consequences of this strong spatial clustering of coinfections.

## Introduction

Coinfections of multiple pathogens can influence epidemiology and disease severity [1]. An understanding of ecological drivers of coinfections is important to improve a targeted public health response. Human infections by zoonotic orthohantaviruses and *Leptospira* spp. are (re-)emerging zoonoses that are almost indistinguishable in their clinical presentation [2] and can often be mistaken for each other.

*Leptospira* spp. are gram-negative bacteria of the class *Spirochaetes*, order *Leptospirales*, family *Leptospiraceae* and are 6–20 μm in size and 0.1 μm in diameter [3]. They can be divided into saprophytic, intermediate and pathogenic groups (including *L. kirschneri*, *L. borgpetersenii* and *L. interrogans*) [4]. Human infections can occur after contact with infected animals or indirectly through contact with contaminated water or soil. The disease course is in most cases asymptomatic or mild, but can progress in some cases after a febrile phase to multiple organ dysfunction [5]. Human incidences vary globally, with amplifying factors (tropical climate, standing water and low sanitation level) being notably absent at higher latitudes [6]. Rodents and shrews are considered as reservoir hosts for zoonotic *Leptospira* spp. with prevalences reaching 50% depending on species and season [4].

Hantaviruses, order *Bunyavirales*, family *Hantaviridae*, are enveloped viruses with a three segmented RNA genome of negative polarity [7]. Depending on the species, orthohantaviruses can cause haemorrhagic fever with renal syndrome (HFRS) or hantavirus cardiopulmonary syndrome. There is an estimated 150 000 cases of HFRS each year, with more than half occurring in China [8]. In Central Europe, Puumala orthohantavirus (PUUV) is the most important hantavirus as reflected by the large number of human cases, in particular during outbreak years. In Germany, the mean incidence is 0.87 per 100 000 inhabitants [9], but it reached 60 per 100 000 inhabitants in the outbreak year 2012 in the districts Göppingen and Heidenheim in Baden-Wuerttemberg [10]. Although the reservoir of PUUV, the bank vole (*Clethrionomys glareolus*), is distributed throughout Germany, PUUV is present only in the southern and western parts of the country [11]. The occurrence of Dobrava-Belgrade orthohantavirus (DOBV), genotype Kurkino, in Germany follows the geographical distribution of its reservoir, the striped field mouse (*Apodemus agrarius*) and is limited to north-eastern and eastern Germany [9, 12]. Finally, Tula orthohantavirus (TULV) is a broadly distributed orthohantavirus with the common vole (*Microtus arvalis*) as reservoir, but was also detected in other closely related species such as the field vole (*Microtus agrestis*), East European vole (*Microtus levis*) and water vole (*Arvicola amphibius*) [13]. TULV is generally considered to be of no (or low) pathogenicity, with only sporadic evidence of human infections [13, 14].

Coinfection with both pathogens has been confirmed in humans and rodents [15, 16] and in this study, we screened rodents and shrews from central Germany over the course of a year for pathogenic *Leptospira* spp., TULV, DOBV and PUUV and evaluated the frequency of dual hantavirus–*Leptospira* infections.

## Material and methods

### Trapping and dissection

**Fig. 1.**
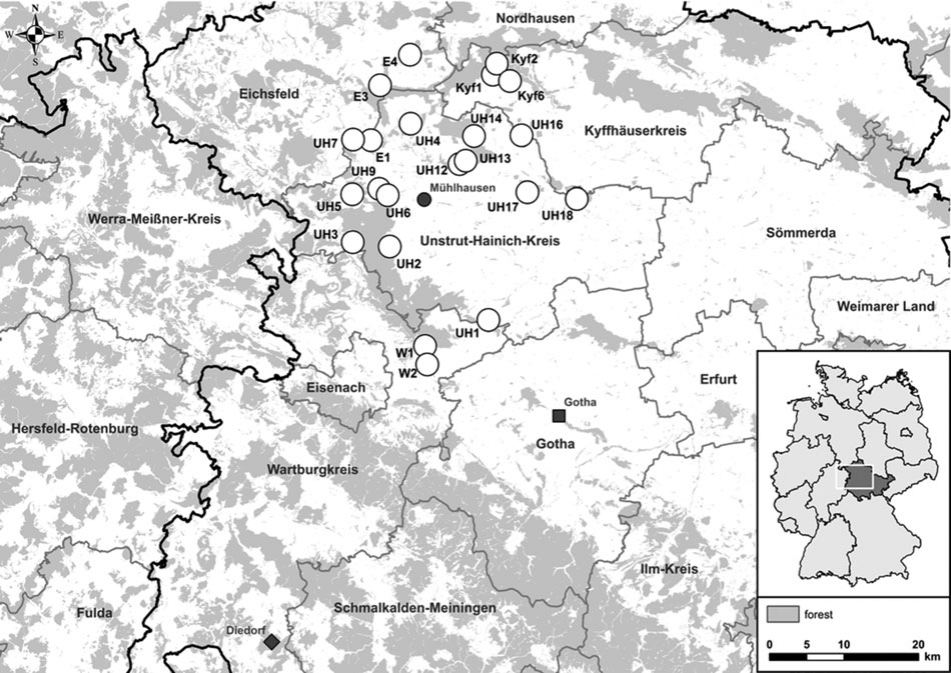
Map of 22 trap sites around Mühlhausen (black circle) in Thuringia, Germany (see small overview map). Additionally, the sites Diedorf (diamond) and Gotha (square) are shown where previously Puumala orthohantavirus (PUUV) and Tula orthohantavirus (TULV) were detected, respectively.

Small mammals were trapped in spring, summer and fall 2017 at 22 sites in Thuringia, central Germany (Fig. 1). In central Germany, the distributional ranges of all abovementioned pathogens and their hosts probably overlap [4, 13, 17]. Each site consisted of perennial grassland as well as the adjacent grassland-forest ecotone. In each of these habitats small mammals were trapped with 36 snap traps (metal snap traps, Deufa, Neuburg, Germany) set in four rows with 10 m trap spacing. In the ecotone, two rows were set in the grassland section and two rows in the transition to the prevailing forest habitat. The trapping at site UH6 was discontinued after spring season due to logistic reasons. All procedures involving animals were conducted according to relevant legislation and by permission of the Thuringia state office of Consumer Protection (permit 22-2684-04-15-105/16). Small mammal carcasses were frozen at −20 °C until dissection. During dissection, small mammals were measured, weighed and sex was determined. To avoid contamination, sterile instruments for each individual were used. Lung and kidney tissue were collected and stored at −20 °C. If necessary, species and sex were determined by corresponding polymerase chain reaction (PCR) assays using kidney tissue-derived DNA as previously described [4, 18].

### *Leptospira* spp. DNA screening

A pin-head-sized piece of kidney tissue was used for DNA extraction by Tissue DNA Kit according to the manual of the manufacturer (Roboklon, Berlin, Germany). DNA concentration was determined with Nanodrop ND-1000 (peqlab Biotechnologie GmbH, Erlangen, Germany). DNA samples were tested in the conventional *lipL32* PCR for the presence of pathogenic leptospires [4, 18]. Genomospecies identification of positive samples was done by *secY* PCR, dideoxy chain termination sequencing of PCR products with BigDye Terminator v1.1 Cycle Sequencing Kit (Applied Biosystems™, Waltham, MA, USA) and sequence comparison to GenBank entries by nucleotide Basic Local Alignment Search Tool (BLASTn) (https://blast.ncbi.nlm.nih.gov/Blast.cgi) [4].

### Hantavirus screening by RT-PCR

RNA was extracted from a lentil-sized piece of lung tissue with QIAZOL reagent (QIAGEN, Hilden, Germany) and eluted in 100 μl DNase/RNase free water (Thermo Fisher Scientific, Schwerte, Germany) [13]. RNA concentration was measured with Nanodrop ND-1000. Reverse transcription-PCR (RT-PCR) was performed using SuperScript™ III One-Step RT-PCR with Platinum Taq-Kit (Invitrogen, Darmstadt, Germany). TULV/PUUV S segment RT-PCR screening used the primer pair PUUV342F and PUUV1102R [19]. DOBV RNA screening was based on RT-PCR using the S segment primer pair D113M and D955CM [20]. RT-PCR products of the expected size were directly sequenced using BigDye Terminator v1.1 Cycle Sequencing Kit (Applied Biosystems™).

### Phylogenetic analysis

ClustalW multiple alignments of obtained nucleotide (nt)-sequences were constructed using BioEdit v7.2.5 [21]. The best fitting substitution model was determined by jModelTest v2.1.6 [22]. Phylogenetic trees were reconstructed according to maximum likelihood and Bayesian algorithms via FasttreeMP v2.1.10 and MrBayes v3.2.6 on CIPRES Science Gateway [23–25]. Subsequently, a consensus tree was established as bootstrap values ≥75 of the maximum likelihood tree were transferred to the Bayesian tree only if branches of both trees were consistent. Probabilities of node support of the Bayesian tree are given when the value was ≥95%.

### Statistical analysis

To estimate key drivers of coinfections, a generalised linear mixed model was generated for *Microtus* spp. in grassland, where the individual coinfection status (binomial variable; TULV RNA positive and *Leptospira* spp. DNA positive) was the dependent variable. Individual demographic variables (sex, weight as a proxy for age) [26] as well as population level variables (TULV prevalence, *Leptospira* spp. prevalence, abundance (trap success as individuals per 100 trap nights), abundance in the previous season and season itself) were fixed factors. Site was incorporated as a random factor. The most appropriate model was determined using a multimodel inference approach. Using the *dredge* function from the *MuMIn*-package all possible combinations of fixed factors were ranked by their conditional Akaike information criterion (AIC). The best fitting models were defined as being within a ΔAIC of <2 of the best model (lowest AIC). Model coefficients were averaged using the *model.avg* function. We present the relative importance for each factor as the sum of Akaike weights in the best fitting models where the respective factor occurs as well as the 95% confidence interval (CI) for each factor. Here, a factor can be considered significant if the CIs do not include zero.

As trap success of *Microtus* spp. in the grassland/forest ecotone precluded a full model, a chi-square test was used to compare the overall prevalence in both habitats. CIs for prevalences were calculated using the *exactci*-function from the PropCIs-package. All analyses were performed using R [27].

## Results

### Small mammal trapping

During 2017, 1758 small mammals were trapped, including 90 striped field mice, 351 yellow-necked mice (*A. flavicollis*), 61 wood mice (*A. sylvaticus*), 11 field voles, 718 common voles, three European pine voles (*M. subterraneus*), 490 bank voles and 34 shrews including three bicoloured white-toothed shrews (*Crocidura leucodon*), 26 common shrews (*Sorex araneus*), two crowned shrews (*S. coronatus*) and three Eurasian pygmy shrews (*S. minutus*) (Table 1).

**Table 1.** Small mammals trapped in Thuringia, Germany, and results of *Leptospira* spp. PCR and hantavirus RT-PCR analyses for Dobrava-Belgrade orthohantavirus (DOBV), Tula orthohantavirus (TULV) and Puumala orthohantavirus (PUUV).

Common name (scientific name)	Total number of animals trapped	*Leptospira* spp. (number of animals tested/total number of animals, prevalence, 95% confidence interval)				Hantavirus (number of animals tested/total number of animals, prevalence, 95% confidence interval)			
		Spring	Summer	Fall	Total	Spring	Summer	Fall	Total
Striped field mouse (*Apodemus agrarius*)	90	0/2	10/53 (18.9%, 9.4–32.0%)	9/31 (29%, 14.2–48.0%)	19/86 (22.1%, 13.9–32.3%)	0/2	0/53	0/31	0/86 No DOBV
Yellow-necked mouse (*Apodemus flavicollis*)	351	0/59	32/154 (20.8%, 14.7–28.0%)	23/132 (17.4%, 11.4–25.0%)	55/345 (15.9%, 12.2–20.2%)	n.d.	n.d.	n.d.	n.d.
Wood mouse (*Apodemus sylvaticus*)	61	1/25 (4%, 0.1–20.4%)	3/15 (20%, 4.3–48.1%)	4/20 (20%, 5.7–43.7%)	8/60 (13.3%, 5.9–24.6%)	n.d.	n.d.	n.d.	n.d.
Field vole (*Microtus agrestis*)	11	0/1	2/5 (40%, 5.3–85.3%)	4/5 (80%, 28.4–99.5%)	6/11 (54.5%, 23.4–83.2%)	0/1	0/5	0/5	0/11
Common vole (*Microtus arvalis*)	718	4/78 (5.1%, 1.4–12.6%)	74/285 (26%, 21.0–31.4%)	127/313 (40.6%, 35.1–46.2%)	205/676 (30.3%, 26.9–33.9%)	16/79 (20.2%, 12.0–30.1%)	26/280 (9.3%, 6.2–13.3%)	51/315 (16.2%, 12.3–20.7%)	93/674 (13.8%, 11.3–16.6%) TULV
European pine vole (*Microtus subterraneus*)	3	0/0	0/2	0/1	0/3	0/0	0/2	0/1	0/3
Bank vole (*Clethrionomys glareolus*)	490	1/112 (0.9%, 0.0–4.9%)	27/158 (17.1%, 11.6–23.9%)	26/204 (12.7%, 8.5–18.1%)	54/474 (11.4%, 8.7–14.6%)	1/111 (1.0%, 0.0–4.9%)	2/157 (1.3%, 0.2–4.5%)	4/203 (2.0%, 0.5–5.0%)	7/471 (1.5%, 0.6–3.0%) PUUV
Bicoloured white-toothed shrew (*Crocidura leucodon*)	3	0/0	0/0	1/3	1/3	n.d.	n.d.	n.d.	n.d.
Common shrew (*Sorex araneus*)	26	0/1	1/6 (16.7%, 0.4–64.1%)	1/19 (5.3%, 0.1–26.0%)	2/26 (7.7%, 0.9–25.1%)	n.d.	n.d.	n.d.	n.d.
Crowned shrew (*Sorex coronatus*)	2	0/0	0/0	0/2	0/2	n.d.	n.d.	n.d.	n.d.
Eurasian pygmy shrew (*Sorex minutus*)	3	0/0	0/0	0/3	0/3	n.d.	n.d.	n.d.	n.d.
Total	1758	6/278 (2.2%, 0.8–4.7%)	149/678 (22%, 18.9–25.2%)	195/733 (26.6%, 23.4–30.0%)	350/1689 (20.7%, 18.8–22.7%)				

n.d., not done.

### *Leptospira* spp. screening

For 1689 of the 1758 trapped small mammals kidney tissue was available. Overall, 350 of 1689 (20.7%) small mammals tested positive in the *lipL*32 PCR (Table 1). In rodents, the overall prevalence varied between species: field voles (54.5%; 6/11, CI 23.4–83.3%), common voles (30.3%; 205/676, CI 26.9–33.9%), striped field mice (22.1%; 19/86, CI 13.9–32.3%), yellow-necked mice (15.9%; 55/345, CI 12.2–20.2%), wood mice (13.3%; 8/60, CI 5.9–24.6%) and bank voles (11.4%; 54/474, CI 8.7–14.6%). Two of 26 common shrews (7.7%; CI 0.9–25.1%) were tested positive and one of three bicoloured white-toothed shrews was also positive. None of the European pine voles, crowned shrews and Eurasian pygmy shrews tested positive.

The overall prevalence increased from spring (2.2%, 6/278, CI 0.8–4.7%) to summer (22%, 149/678, CI 18.9–25.2%) and fall (26.6%, 195/733, CI 23.4–30.0%). *Leptospira* spp. were detected at 21 of 22 sites with an average site-specific prevalence ranging from 2.4% (2/84, CI 0.3–8.4%) at site UH3 to up to 41.5% (22/53, CI 28.1–55.9%) at site W1. The highest prevalence was measured at site E4 with 76.5% (13/17, CI 50–93.2%) in fall just for common voles. The most abundant genomospecies was *L. kirschneri* (*n* = 108; 93.1%); *L. borgpetersenii* was found only in a few individuals (*n* = 8, 6.9%); no other genomospecies was identified. Common voles only harboured *L. kirschneri* (*n* = 92; 100%). Similarly, in striped-field mice (*n* = 2), wood mice (*n* = 2), field voles (*n* = 1) and common shrews (*n* = 1) also exclusively *L. kirschneri* was identified. Yellow-necked mice carried *L. kirschneri* (62.5%; 6/9) or *L. borgpetersenii* (37.5%, 3/9), and bank voles also carried *L. kirschneri* (45%, 4/9) or *L. borgpetersenii (*55.5%, 5/9). *L. kirschneri* and *L. borgpetersenii* circulated in the same bank vole population at one site (KYF1) during the same trapping season. Otherwise only a single *Leptospira* genomospecies was detected per site depending on trapping location and species.

### Hantavirus screening

**Fig. 2.**
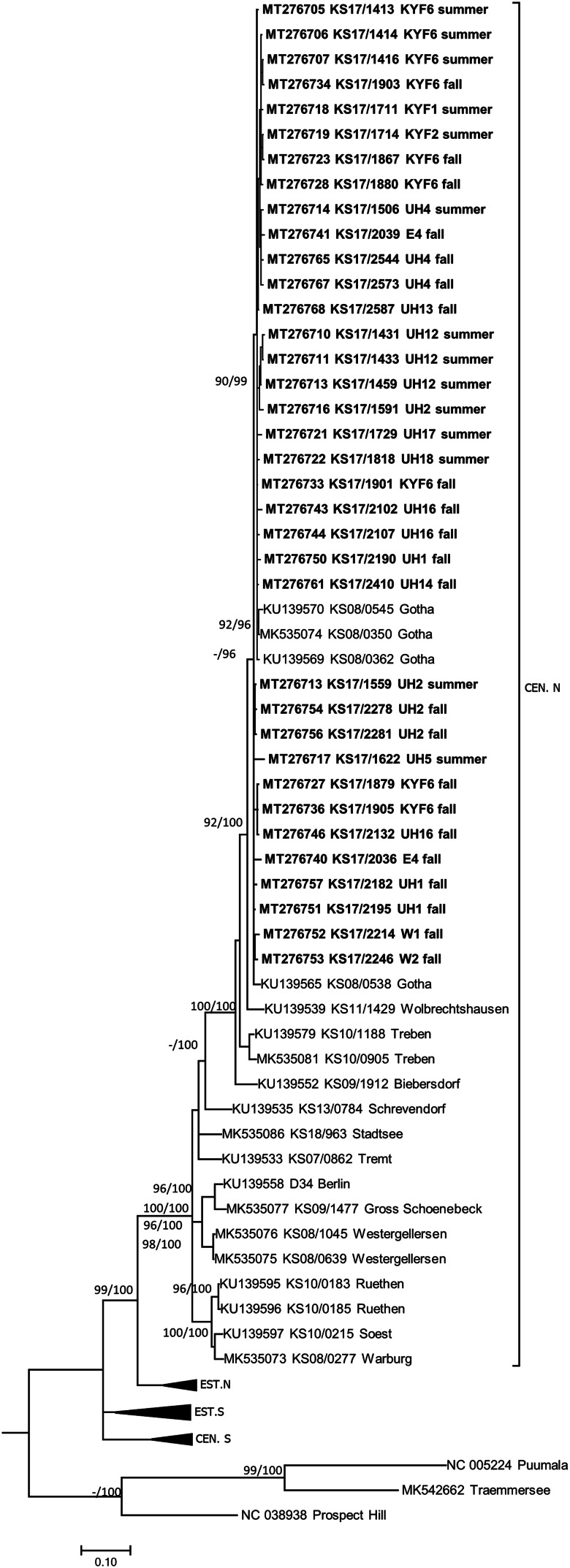
Consensus phylogenetic tree of the partial S segment sequences of Tula orthohantavirus (TULV) (alignment length 549 nucleotides (nt), positions 406–951, counting according to TULV S segment, accession number NC_005227). TULV is sorted in the clades Central North (CEN.N), Central South (CEN.S), Eastern North (EST.N) and Eastern South (EST.S). The consensus tree is based on Bayesian analyses with 10^7^ generations and a burn-in phase of 25%, and maximum-likelihood analyses, with 1000 bootstraps and 50% cut-off using the general time reversible (GTR) substitution model with invariant sites and a gamma distributed shape parameter for both algorithms. Posterior probabilities exceeding 95% from Bayesian analyses are given behind and bootstrap values are given before the slash for major nodes if exceeding 75%. The tree reconstructions were done via CIPRES [23]. Alignments were constructed with Bioedit V7.2.3. [21] using the Clustal W Multiple Alignment algorithm implemented in the program. Names in bold indicate newly generated sequences (see Supplementary Table S1). Triangles indicate compressed branches (see Supplementary Table S2 for used sequences). Clade designation followed previous publications for TULV [28].

TULV-RNA was detected in 13.8% (93/674, CI 11.3–16.6%) of common voles, in none of the 11 field voles and none of the three European pine voles (Table 1). Overall prevalence in common voles was highest in spring with 20.2% (16/79, CI 12.0–30.1%), followed by fall with 16.2% (51/315, CI 12.3–20.7%) and summer with 9.3% (26/280, CI 6.2–13.3%). No TULV-RNA was found at three sites (E3, UH3, UH9; combined 0/24, CI 0.0–14.2%), while prevalences of up to 33.8% (KYF6; 23/68, CI 17.8–37.4%) were detected among sites where at least 10 common voles were tested. The highest prevalence from sites with 10 or more tested common voles was measured in spring at site UH17 with 58.3% (7/12, CI 27.7–84.8%). TULV RNA positive voles originated from 18 of 21 sites where common voles were trapped. TULV was present at only four sites in spring, despite common vole presence at 15 sites. The overall prevalence at these four sites was 50% (16/32, CI 38.9–68.1%). In summer, TULV was present at 14 sites and at 15 sites in fall. The four sites with high prevalences in spring did not differ significantly from the rest in summer (*χ*^2^ = 0.031, *P* = 1) or in autumn (*χ*^2^ = 0.474, *P* = 0.57). Phylogenetic analysis showed that the sequences clustered with TULV sequences from geographically close Gotha, Thuringia, Germany (Fig. 1, square), in the TULV Central North (CEN. N) clade (Fig. 2).

**Fig. 3.**
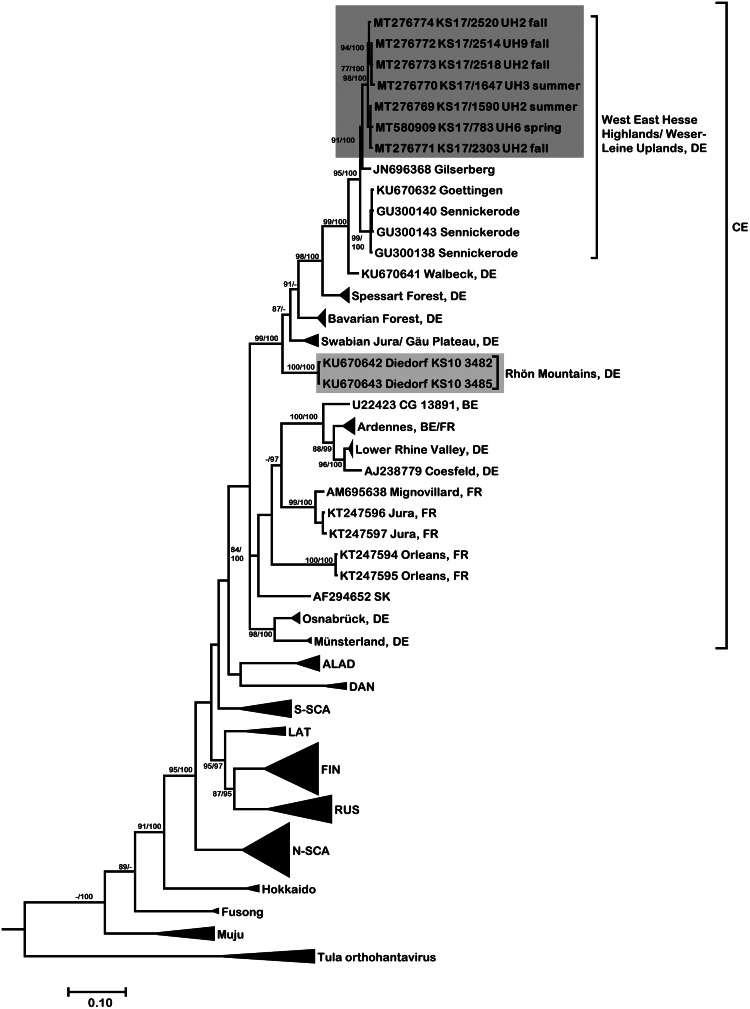
Consensus phylogenetic tree of partial S segment sequences for Puumala orthohantavirus (PUUV) (alignment length 711 nt, positions 355–1065, counting according to PUUV S segment, accession number NC_005224). PUUV is sorted in the clades Alpe-Adrian (ALAD), Central European (CE) clade including Belgium (BE), France (FR), Germany (DE), Slovakia (SK), Danish (DAN), Finnish (FIN), Latvian (LAT), Northern-Scandinavian (N-SCA), Russian (RUS), Southern-Scandinavian (S-SCA) as well as the PUUV strains Hokkaido, Muju and Fusong. The consensus tree is based on Bayesian analyses with 1.5 × 10^7^ generations and a burn-in phase of 25%, and maximum-likelihood analyses, with 1000 bootstraps and 50% cut-off using the general time reversible (GTR) substitution model with invariant sites and a gamma distributed shape parameter for both algorithms. Posterior probabilities exceeding 95% from Bayesian analyses are given behind and bootstrap values are given before the slash for major nodes if exceeding 75%. The tree reconstructions were done via CIPRES [23]. Alignments were constructed with Bioedit V7.2.3. [21] using the Clustal W Multiple Alignment algorithm implemented in the program. Names in bold indicate newly generated sequences (see Supplementary Table S1). Triangles indicate compressed branches (see Supplementary Table S2 for used sequences). Clade designation followed previous publications for PUUV [11, 29].

In 1.5% (7/471, CI 0.6–3.0%) of tested bank voles PUUV-RNA was detected. Positive voles were trapped at neighbouring sites UH2, UH3, UH9 and UH6 (Fig. 1). Phylogenetic analysis revealed that the novel PUUV strains belong to the PUUV Central European (CE) clade. The novel sequences clustered closest to sequences from western and northwestern parts of Germany such as Gilserberg (Hesse), Goettingen and Sennickerode (both in Lower Saxony) (Fig. 3). Interestingly, PUUV sequences from Diedorf (Thuringia, Fig. 1, diamond), a site only 50 km away from the trapping locations in this study (Fig. 1), clustered differentially, i.e. with sequences from southern Germany, like Swabian Jura and Bavarian forest.

DOBV infection was not detected in any of the 86 tested striped field mice (Table 1).

### Coinfections

In 6.6% (44/671, CI 4.8–8.7%) of common voles, we detected a coinfection of *Leptospira* spp. with TULV. There was no statistical difference between coinfection prevalence detected in forest ecotone (7.7%; 3/39, CI 1.6–20.9%) and in grassland (6.5%; 41/632, CI 4.7–8.7%) (*χ*^2^ = 0.0114, *P* = 0.91). Seasonal differences became apparent. While the prevalence of common voles infected with both pathogens differed significantly (*χ*^2^ = 6.563, *P* = 0.01) between summer 4.3% (CI 2.2–7.4%, 12/280) and fall 10.2% (CI 7.1–14.1%, 32/313), no coinfections were detected in spring (0/78).

**Fig. 4.**
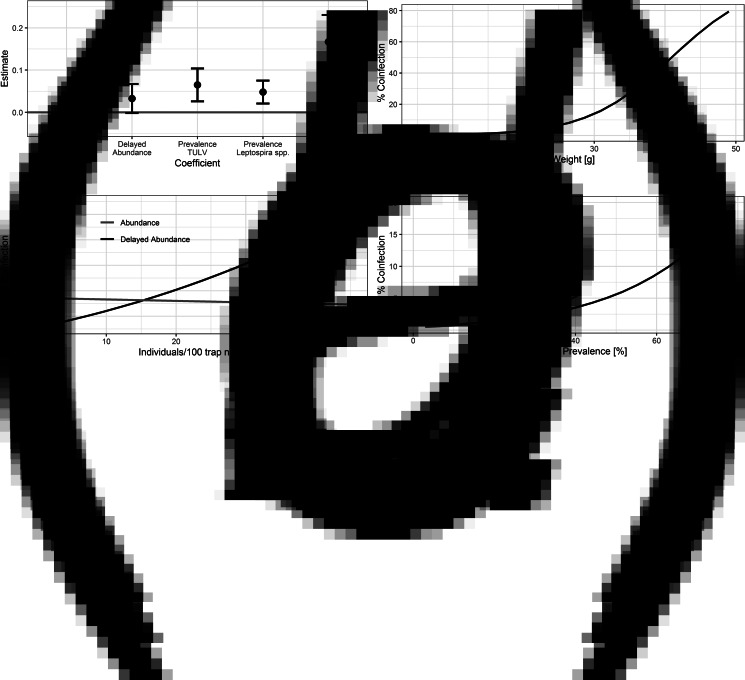
Graphical representation of the model averaging following multimodel inference. (a) Averaged factor mean estimates and their 95% confidence interval. (b–d) Prediction for each factor in the average model. For each predicted factor all other factors were kept constant at their respective mean value. (b) Relationship between individual weight and prevalence of coinfections. (c) Density dependence (direct and delayed) of coinfections. (d) Relationship between single pathogen infections and the prevalence of coinfections.

The initial global generalised linear mixed model had a *R*^2^_marginal_ of 0.52 and no overdispersion, but the factor *season* was associated with increased multicollinearity (variance inflation factor >4) and was subsequently omitted from the model. Table 2 shows the comparison of candidate models as well as their respective AIC and model weights. The first three models were included in the AIC cut-off value of Δ2 and subject to model averaging. Averaged parameter estimates and respective relative importance are presented in Table 3. Individual coinfection probability with TULV and *Leptospira* spp. was driven by both, individual and population-level factors. Individual age and population-level TULV and *Leptospira* spp. prevalences are significant factors, in determining coinfections. Both abundance measures (delayed and direct) were selected in the averaging process, but only the abundance in the previous season seemed to influence subsequent coinfection dynamics (delayed density dependence). Parameter effect sizes (mean and 95% CI) are shown in Figure 4a. Individual weight had the most dominant effect, while the CIs of the delayed abundance marginally incorporated zero. Model predictions for each factor are shown in Figure 4 (b, c), where for each factor all other factors were kept constant at their respective mean value. Predictions show that older individuals have a higher probability of being coinfected and that a higher abundance of common voles in the previous season increased the probability of subsequent individual coinfections. For both pathogens an increasing prevalence (while keeping the other pathogen constant) increased the probability of coinfections. As both pathogens differ in their range of detected prevalences, this effect is more prominent in *Leptospira* spp. compared to TULV. However, the relationship between the increase in prevalences of single pathogens and coinfections is significantly better explained by an exponential increase (*Leptospira* spp.: *R*^2^ = 0.99; TULV: *R*^2^ = 0.99) compared to a linear one (*Leptospira* spp.: *R*^2^ = 0.86; TULV: *R*^2^ = 0.90) (comparison *Leptospira* spp.: *F* = 334.88; *P* < 0.001, TULV: *F* = 451.06; *P* < 0.001). This indicates that prevalences near the upper end of the potential range result in disproportionally more coinfections compared to lower prevalences. Two of 469 bank voles (0.4%) tested positive for PUUV and *Leptospira* spp. These originated both from site UH2 in fall.

**Table 2.** Binomial generalised linear models explaining the probability of the occurrence of coinfections between *Leptospira* spp. and TULV. Estimates of continuous variables and presence of categorical (indicated by+) population-level and individual variables are presented. Models with Δ AIC >2 were excluded. DF = degrees of freedom, logLik = log-likelihood value

Epidemiological		Ecological								
Population				Individual						
Prevalence TULV	Prevalence *Leptospira* spp.	Abundance	Delayed abundance	Sex	Weight	df	logLik	AICc	*ΔAICc*	Model weight
0.062	0.047		0.033		0.173	6	−93.424	199.1	0	0.343
0.069	0.051				0.151	5	−95.104	200.4	1.3	0.179
0.067	0.046	−0.008	0.033		0.173	7	−93.321	200.9	1.87	0.135
0.062	0.047		0.033	+	0.173	7	−93.392	201.1	2.01	0.126
0.072	0.050	−0.004			0.151	6	−95.068	202.3	3.29	0.066
0.070	0.051			+	0.151	6	−95.085	202.4	3.32	0.065
0.068	0.046	−0.008	0.033	+	0.173	8	−93.291	202.9	3.89	0.049
0.073	0.050	−0.004		+	0.151	7	−95.052	204.4	5.33	0.024
	0.063		0.039		0.161	5	−99.073	208.3	9.24	0.003
	0.066	0.021	0.036		0.163	6	−98.118	208.4	9.39	0.003
0.095			0.049		0.159	5	−99.844	209.8	10.78	0.002
	0.063		0.039	+	0.162	6	−99.071	210.4	11.29	0.001
	0.066	0.021	0.036	+	0.163	7	−98.117	210.5	11.46	0.001
0.102		−0.016	0.046		0.160	6	−99.618	211.4	12.39	0.001
0.095			0.049	+	0.160	6	−99.689	211.6	12.53	0.001
	0.075	0.025			0.139	5	−100.857	211.9	12.81	0.001
	0.070				0.137	4	−102.131	212.4	13.3	0

**Table 3.** Model averaged estimates for the probability of the occurrence of coinfections between *Leptospira* and TULV. Relative importance as the sum of Akaike weights of all best fitting model where the specific variable is included. Significant factors are highlighted in bold. S.E. = Standard Error

Variable	Estimate	S.E.	*z*-value	*P*-value	Relative importance
Intercept	−10.126	1.425	7.086	**<0**.**001**	
Prevalence TULV	0.065	0.020	3.264	**0**.**001**	1.00
Prevalence *Leptospira* spp.	0.048	0.014	3.484	**<0**.**001**	1.00
Weight	0.167	0.032	5.143	**<0**.**001**	1.00
Delayed abundance	0.033	0.018	1.864	0.062	0.73
Abundance	−0.008	0.018	0.448	0.654	0.21

## Discussion

We detected *Leptospira* spp. in several small mammal species in central Germany. Compared to a previous study in the same region (Fig. 1, square) [4], overall prevalence was higher in this study. However, the tendency that *Microtus* spp. had, on average, higher prevalence compared to most other species is mirrored here. In a European context, studies that screened at least 10 individuals of one species, generally reported similar prevalence for striped field mice (12.0–19.6%) [4, 16, 31], common voles (14.0–30.0%) [4, 16, 31–33] and field voles (12.0–30.1%) [4, 31]. For the yellow-necked mouse only a study in Serbia detected a higher average prevalence with 34.3% [33], for the wood mouse studies detected similar average prevalence with 15.4% and 18.0% [31, 34] and for bank voles our study is in line with a previously published prevalence [4, 31].

In general, prevalence increased from spring to fall, likely reflecting more favourable conditions for survival outside the host at high temperature and in moist soil [3]. Interestingly, the strong variance of *Leptospira* spp. prevalence was not only dependent on season but also on site. In fall, the season with the highest overall prevalence, there was high spatial variability in *Leptospira* spp. prevalence. While there was no *Leptospira* spp. at some sites, three sites exhibited >65% prevalence for the common vole in fall (E4, KYF6, W2). A comparable *Leptospira* spp. prevalence is often reported only in Norway rats (*Rattus norvegicus*) collected in sewage systems [35].

High prevalence of *Leptospira* spp. in certain sites arise from local environmental conditions such as soil composition (e.g. mineral and salt composition), soil humidity [36] and the presence of water bodies. Irrigation can be a significant factor for *Leptospira* prevalence in rodents [37] and human outdoor activity, mainly watersports, is related to localised outbreaks of leptospirosis in humans [38, 39]. The effect of livestock on human or even rodent infection risk is still unclear [40] and requires further investigation. On a larger scale, weather effects like intense rainfall with subsequent flooding have been shown to cause more widespread outbreaks of leptospirosis [39]. Further studies should incorporate these risk factors to estimate the spatial persistence of *Leptospira* in their natural reservoirs.

In grassland, prevalence was especially high in common and field voles, which were exclusively infected with *L. kirschneri* [4, 31, 41]. Forest rodents were found to carry either *L. kirschneri* or *L. borgpetersenii*; *L. interrogans* was not detected here. We detected *L. kirschneri* in wood mice and either *L. kirschneri* or *L. borgpetersenii* in bank voles and yellow-necked mice. Other studies reported lower prevalence for *L. borgpetersenii* but high prevalence for *L. interrogans* in forest rodents [4, 31, 41]. All these studies are consistent with our finding that *L. kirschneri* is the most frequently found *Leptospira* genomospecies in small mammals in Germany [4, 31, 41].

The detection of TULV-RNA at 18 of 21 sites in this study where common voles were trapped is in line with the German-wide distribution of this pathogen [13]. The overall prevalence of 13.9% in common voles is comparable to previously published values of 6.2–23.4% in Europe including Austria, Czech Republic, France, Germany and Hungary [13, 16, 28, 32]. Field voles and European pine voles were not infected with TULV, even though TULV-positive common voles were present in the sites. This finding confirms the common vole to be the main reservoir for TULV and other *Microtus* spp. to be rather accidental hosts [13] even though it is based on a small number of individuals from these two species that were available for analyses. As expected, the sequences clustered in the CEN.N clade of TULV together with sequences from geographically close origin (see [28]).

The very low prevalence of PUUV in this study was most likely a result of the study location at the distributional edge of this hantavirus in Germany. High PUUV prevalence was detected earlier in bank voles during the hantavirus outbreak year 2010 in the western part of Thuringia. Those published PUUV sequences (site Diedorf, see Fig. 1) formed a separate clade ‘Rhön Mountains’ [11, 30]. Thuringia is situated at the eastern distribution border of PUUV in Germany [11] and features zones with previously reported disease clusters in humans and infected bank voles only in the western part of the state [9, 11, 30], while the exact extent of the distributional range is largely unknown. The presented phylogeny provides further information on the dynamics of PUUV in bank voles along its distribution border, as sequences from this study did not cluster with sequences from the abovementioned site Diedorf in Thuringia, but instead with sequences from Lower Saxony and Hesse. This observation may suggest two immigration routes of PUUV-infected bank voles into Thuringia over time, which presents an interesting opportunity to study the short- and long-term dynamics of zoonotic pathogens along the edges of their distributional range in the future.

In this study, we did not detect DOBV infections in 86 striped field mice. DOBV infections have been detected only in striped field mice from more eastern and northern located sites, including the eastern part of Thuringia [12, 42]. Likewise, human infections were detected exclusively in eastern and north-eastern Germany [9, 43].

Coinfection with both, *Leptospira* and TULV in common voles were observed before in Hungary with a prevalence of 3.7% [16]. We identified both, individual and population-level factors associated with coinfection of *Leptospira* and hantavirus in common voles. Individual-level drivers seemed to be mostly associated with age. For each pathogen this has been previously described [4, 35, 44]. The possibility of infection increases over each individual's lifetime and common voles are probably persistently infected with both pathogens, although we have to acknowledge that weight might be an imperfect proxy for age, especially when chronically infected, coinfected individuals could potentially suffer from malnourishment.

Overall, coinfections of *Leptospira* spp. and TULV did depend on host density. Rather than coinfections increasing with immediate density, there was a time-lagged response, where individual coinfections were positively correlated to the density 3 months ago. For other pathogens, this time delay has been shown to be an integral part of the transmission process where an increase in density enhances the availability of susceptible hosts that later can become infected [45]. In coinfections, this aspect might even be amplified, as the transmission process for two pathogens has to be completed. The route of transmission can potentially add to the delayed effect. Rodriguez-Pastor *et al*. [46] detected delayed density dependence in *Bartonella rochalimae* and attributed it to the flea life-cycle as a potential cause for the delayed response. In our context, *Leptospira* spp. can survive outside of their host up to 9 weeks in soil [47] and up to 20 months in freshwater [48, 49]. Long periods of environmental survival might preclude any association with immediate host abundance and rather favour delayed responses.

Unsurprisingly, both pathogens are positively associated with increased coinfections, representing the underlying mathematical probability of coinfections to occur when prevalences of both pathogens increase. However, this relationship is best characterised by an exponential regression rather than a linear one (Fig. 4d), indicating that high prevalences are associated with disproportionally more coinfections. This could be interpreted as increased availability of individuals susceptible to coinfections in high prevalence scenarios for both pathogens. Telfer *et al*. [50] highlighted the importance of pathogen community interaction in determining the overall individual susceptibility to subsequent infections. This would imply that an infection with one of the two pathogens would compromise immunocompetency of the infected individual facilitating a ‘more efficient’ infection with the other pathogen. Our methodology is, however, not suitable to track individual changes within a population across time and might therefore miss subtle individual effects.

Consequently, frequent coinfections were observed in areas where a particularly high prevalence of *Leptospira* spp. was detected. We conclude that, at least for TULV in grassland, high levels of coinfections with *Leptospira* spp. are rather driven by the spatial assemblage of high *Leptospira* spp. prevalences than by TULV prevalence. Despite the low zoonotic potential of TULV [51], coinfections are of general concern. At sites with a high prevalence of *Leptospira* spp. in rodents and an associated increase in human leptospirosis cases, our results suggest that there is also an increased risk of hantavirus coinfections, that might go undetected in humans when coinfections exhibit similar clinical presentations. The spatial assemblage of high *Leptospira* spp. prevalence is therefore of concern as it might also present hot-spots for coinfections with other pathogens. The environmental and epidemiological drivers associated with the patchy occurrence of those hot-spots should be the topic of future research.

## Data Availability

The data for the study are available from the corresponding author.

## Supplementary material

For supplementary material accompanying this paper visit http://dx.doi.org/10.1017/S0950268821000443. click here to view supplementary material
