# Supplementary material for: Hantavirus–Leptospira coinfections in small mammals from central Germany
Source: Epidemiol Infect. 2021 Feb 22;149:e97. doi: 10.1017/S0950268821000443 (PMC8101269; doi:10.1017/S0950268821000443)
Supplement: Supplementary file 1 [file S0950268821000443sup001.docx]

Supplementary Table S1 List of all hantavirus sequences generated during this study.

| Sample ID | Species | Date captured | Area | Hantavirus | Accession number | Sequence identical to |
| --- | --- | --- | --- | --- | --- | --- |
| KS17/1137 | *M. arvalis* | 10.08.2017 | E1 | TULV | MT276704 |  |
| KS17/2036 | *M. arvalis* | 23.10.2017 | E4 | TULV | MT276740 | MT276742 |
| KS17/2039 | *M. arvalis* | 24.10.2017 | E4 | TULV | MT276741 |  |
| KS17/2043 | *M. arvalis* | 25.10.2017 | E4 | TULV | MT276742 | MT276740 |
| KS17/1711 | *M. arvalis* | 17.08.2017 | KYF1 | TULV | MT276718 | MT276739 |
| KS17/1963 | *M. arvalis* | 24.10.2017 | KYF1 | TULV | MT276739 | MT276718 |
| KS17/1714 | *M. arvalis* | 17.08.2017 | KYF2 | TULV | MT276719 | MT276720 |
| KS17/1716 | *M. arvalis* | 17.08.2017 | KYF2 | TULV | MT276720 | MT276719 |
| KS17/1413 | *M. arvalis* | 16.08.2017 | KYF6 | TULV | MT276705 | MT276708 |
| KS17/1414 | *M. arvalis* | 16.08.2017 | KYF6 | TULV | MT276706 | MT276724, MT276731, MT276725, MT276729, MT276730, MT276731, MT276732, MT276737, MT276738, MT276762 |
| KS17/1416 | *M. arvalis* | 16.08.2017 | KYF6 | TULV | MT276707 | MT276709, MT276763, MT276763 |
| KS17/1417 | *M. arvalis* | 16.08.2017 | KYF6 | TULV | MT276708 | MT276705 |
| KS17/1420 | *M. arvalis* | 16.08.2017 | KYF6 | TULV | MT276709 | MT276707, MT276763, MT276763 |
| KS17/1867 | *M. arvalis* | 26.10.2017 | KYF6 | TULV | MT276723 | MT276726 |
| KS17/1874 | *M. arvalis* | 24.10.2017 | KYF6 | TULV | MT276725 | MT276706, MT276724, MT276731, MT276725, MT276729, MT276730, MT276731, MT276732, MT276737, MT276738, MT276762 |
| KS17/1878 | *M. arvalis* | 24.10.2017 | KYF6 | TULV | MT276726 | MT276723 |
| KS17/1879 | *M. arvalis* | 24.10.2017 | KYF6 | TULV | MT276727 |  |
| KS17/1880 | *M. arvalis* | 24.10.2017 | KYF6 | TULV | MT276728 |  |
| KS17/1881 | *M. arvalis* | 24.10.2017 | KYF6 | TULV | MT276729 | MT276706, MT276724, MT276731, MT276725, MT276730, MT276731, MT276732, MT276737, MT276738, MT276762 |
| KS17/1882 | *M. arvalis* | 24.10.2017 | KYF6 | TULV | MT276730 | MT276706, MT276724, MT276731, MT276725, MT276729, MT276731, MT276732, MT276737, MT276738, MT276762 |
| KS17/1886 | *M. arvalis* | 25.10.2017 | KYF6 | TULV | MT276724 | MT276706, MT276731, MT276725, MT276729, MT276730, MT276731, MT276732, MT276737, MT276738, MT276762 |
| KS17/1888 | *M. arvalis* | 25.10.2017 | KYF6 | TULV | MT276731 | MT276706, MT276724, MT276725, MT276729, MT276730, MT276731, MT276732, MT276737, MT276738, MT276762 |
| KS17/1889 | *M. arvalis* | 25.10.2017 | KYF6 | TULV | MT276732 | MT276706, MT276724, MT276731, MT276725, MT276729, MT276730, MT276731, MT276737, MT276738, MT276762 |
| KS17/1901 | *M. arvalis* | 26.10.2017 | KYF6 | TULV | MT276733 |  |
| KS17/1903 | *M. arvalis* | 26.10.2017 | KYF6 | TULV | MT276734 | MT276735 |

Supplementary Table S1 (continued)

| Sample ID | Species | Date captured | Area | Hantavirus | Accession number | Sequence identical to |
| --- | --- | --- | --- | --- | --- | --- |
| KS17/1904 | *M. arvalis* | 26.10.2017 | KYF6 | TULV | MT276735 | MT276734 |
| KS17/1905 | *M. arvalis* | 26.10.2017 | KYF6 | TULV | MT276736 |  |
| KS17/1911 | *M. arvalis* | 26.10.2017 | KYF6 | TULV | MT276737 | MT276706, MT276724, MT276731, MT276725, MT276729, MT276730, MT276731, MT276732, MT276737, MT276738, MT276762 |
| KS17/1912 | *M. arvalis* | 26.10.2017 | KYF6 | TULV | MT276738 | MT276706, MT276724, MT276731, MT276725, MT276729, MT276730, MT276731, MT276732, MT276737, MT276738, MT276762 |
| KS17/2182 | *M. arvalis* | 27.10.2017 | UH1 | TULV | MT276747 | MT276748, MT276749 |
| KS17/2184 | *M. arvalis* | 28.10.2017 | UH1 | TULV | MT276748 | MT276747, MT276749 |
| KS17/2185 | *M. arvalis* | 28.10.2017 | UH1 | TULV | MT276749 | MT276747, MT276748 |
| KS17/2190 | *M. arvalis* | 29.10.2017 | UH1 | TULV | MT276750 |  |
| KS17/2195 | *M. arvalis* | 29.10.2017 | UH1 | TULV | MT276751 |  |
| KS17/744 | *M. arvalis* | 13.05.2017 | UH2 | TULV | MT276692 |  |
| KS17/1559 | *M. arvalis* | 13.08.2017 | UH2 | TULV | MT276715 | MT276760 |
| KS17/1591 | *M. arvalis* | 11.08.2017 | UH2 | TULV | MT276716 |  |
| KS17/2278 | *M. arvalis* | 28.10.2017 | UH2 | TULV | MT276754 | MT276755, MT276757, MT276759 |
| KS17/2280 | *M. arvalis* | 28.10.2017 | UH2 | TULV | MT276755 | MT276754, MT276757, MT276759 |
| KS17/2281 | *M. arvalis* | 28.10.2017 | UH2 | TULV | MT276756 | MT276758 |
| KS17/2282 | *M. arvalis* | 28.10.2017 | UH2 | TULV | MT276757 | MT276754, MT276755, MT276759 |
| KS17/2284 | *M. arvalis* | 29.10.2017 | UH2 | TULV | MT276758 | MT276756 |
| KS17/2287 | *M. arvalis* | 29.10.2017 | UH2 | TULV | MT276759 | MT276754, MT276755, MT276757 |
| KS17/2292 | *M. arvalis* | 29.10.2017 | UH2 | TULV | MT276760 | KS17/1559 |
| KS17/1506 | *M. arvalis* | 12.08.2017 | UH4 | TULV | MT276714 |  |
| KS17/2532 | *M. arvalis* | 18.10.2017 | UH4 | TULV | MT276763 | MT276707, MT276709,  MT276764 |
| KS17/2539 | *M. arvalis* | 19.10.2017 | UH4 | TULV | MT276764 | MT276707, MT276709, MT276763 |
| KS17/2544 | *M. arvalis* | 20.10.2017 | UH4 | TULV | MT276765 | MT276766 |
| KS17/2545 | *M. arvalis* | 20.10.2017 | UH4 | TULV | MT276766 | MT276765 |
| KS17/2573 | *M. arvalis* | 20.10.2017 | UH4 | TULV | MT276767 |  |
| KS17/788 | *M. arvalis* | 09.05.2017 | UH5 | TULV | MT276693 | MT276694, MT276695 |
| KS17/789 | *M. arvalis* | 09.05.2017 | UH5 | TULV | MT276694 | MT276693, MT276695 |
| KS17/792 | *M. arvalis* | 10.05.2017 | UH5 | TULV | MT276695 | MT276693, MT276694 |
| KS17/793 | *M. arvalis* | 10.05.2017 | UH5 | TULV | MT276696 | MT276697, MT276717 |
| KS17/794 | *M. arvalis* | 10.05.2017 | UH5 | TULV | MT276697 | MT276696, MT276717 |
| KS17/1622 | *M. arvalis* | 11.08.2017 | UH5 | TULV | MT276717 | MT276696, MT276697 |
| KS17/781 | *M. arvalis* | 10.05.2017 | UH7 | TULV | MT276691 |  |
| KS17/1431 | *M. arvalis* | 15.08.2017 | UH12 | TULV | MT276710 |  |
| KS17/1433 | *M. arvalis* | 15.08.2017 | UH12 | TULV | MT276711 | MT276712 |
| KS17/1457 | *M. arvalis* | 13.08.2017 | UH12 | TULV | MT276712 | MT276711 |
| KS17/1459 | *M. arvalis* | 13.08.2017 | UH12 | TULV | MT276713 |  |
| KS17/2587 | *M. arvalis* | 22.10.2017 | UH13 | TULV | MT276768 |  |
| KS17/2410 | *M. arvalis* | 18.10.2017 | UH14 | TULV | MT276761 |  |

Supplementary Table S1 (continued)

| Sample ID | Species | Date captured | Area | Hantavirus | Accession number | Sequence identical to |
| --- | --- | --- | --- | --- | --- | --- |
| KS17/2418 | *M. arvalis* | 20.10.2017 | UH14 | TULV | MT276762 | MT276706, MT276724, MT276731, MT276725, MT276729, MT276730, MT276731, MT276732, MT276737, MT276738 |
| KS17/2102 | *M. arvalis* | 26.10.2017 | UH16 | TULV | MT276743 |  |
| KS17/2107 | *M. arvalis* | 26.10.2017 | UH16 | TULV | MT276744 | MT276745 |
| KS17/2131 | *M. arvalis* | 28.10.2017 | UH16 | TULV | MT276745 | MT276744 |
| KS17/2132 | *M. arvalis* | 26.10.2017 | UH16 | TULV | MT276746 |  |
| KS17/875 | *M. arvalis* | 11.05.2017 | UH17 | TULV | MT276698 | MT276701, MT276702 |
| KS17/878 | *M. arvalis* | 12.05.2017 | UH17 | TULV | MT276699 | MT276700, MT276703, MT276721 |
| KS17/879 | *M. arvalis* | 12.05.2017 | UH17 | TULV | MT276700 | MT276699, MT276703, MT276721 |
| KS17/880 | *M. arvalis* | 12.05.2017 | UH17 | TULV | MT276701 | MT276698, MT276702 |
| KS17/881 | *M. arvalis* | 12.05.2017 | UH17 | TULV | MT276702 | MT276698, MT276701 |
| KS17/882 | *M. arvalis* | 12.05.2017 | UH17 | TULV | MT276703 | MT276699, MT276700, MT276721 |
| KS17/1729 | *M. arvalis* | 17.08.2017 | UH17 | TULV | MT276721 | MT276699, MT276700, MT276703 |
| KS17/1818 | *M. arvalis* | 17.08.2017 | UH18 | TULV | MT276722 |  |
| KS17/2214 | *M. arvalis* | 29.10.2017 | W1 | TULV | MT276752 |  |
| KS17/2246 | *M. arvalis* | 28.10.2017 | W2 | TULV | MT276753 |  |
| KS17/783 | *C. glareolus* | 09.05.2017 | UH6 | PUUV | MT580909 |  |
| KS17/1590 | *C. glareolus* | 11.08.2017 | UH2 | PUUV | MT276769 |  |
| KS17/2303 | *C. glareolus* | 29.10.2017 | UH2 | PUUV | MT276771 |  |
| KS17/1647 | *C. glareolus* | 11.08.2017 | UH3 | PUUV | MT276770 |  |
| KS17/2514 | *C. glareolus* | 24.10.2017 | UH9 | PUUV | MT276772 |  |
| KS17/2518 | *C. glareolus* | 27.10.2017 | UH2 | PUUV | MT276773 |  |
| KS17/2520 | *C. glareolus* | 28.10.2017 | UH2 | PUUV | MT276774 |  |

Supplementary Table S2 List of all sequences used in condensed clades in Fig. 2 and Fig. 3. Lineage nomenclature after [1,2].

| Hantavirus | Clade/strain | Accession number |
| --- | --- | --- |
| Tula orthohantavirus | Central South (CEN.S) | MK386133  MK386134  MK386135  MK386138  MK386139 |
|  | Eastern North (EST.N) | AF063892  KU139555  KU139563  MK535078  MK535083 |
|  | Eastern South (EST.S) | MK386130  MK386131  MK386132  MK386136  MK386137  MK386140  Z69991 |
| Puumala orthohantavirus | Alpe-Adrian (ALAD) | AJ314600  AJ314601  AJ888751  AJ888752  FN37782a  FN377822  KC676609  KC676613 |
|  | CE (Central European) | Ardennes:  AJ277031 - AJ277076, AJ277030, KT247593  Bavarian Forest:  AY954722, DQ016432, EU439968, EU439969, EU439972, JN696374 - JN696376  Lower Rhine Valley:  DQ408268, DQ408271 - DQ408275, KU670633  Münsterland:  JN696362, L36943  Osnabrück:  JN696355, JN696358, KU670635, KU670640, KJ994776  Spessart Forest:  EU246962, JN696356, JN696359, JN696367, JN696371 - JN696373  Swabian Jura/ Gäu Plateaus  DQ094844, EU085558, EU085563, EU085565, JN696361, KU670631 |

Supplementary Table S2 (continued)

| Hantavirus | Clade/strain | Accession number |
| --- | --- | --- |
| Puumala ortohantavirus | Danish  (DAN) | AJ238791  AJ238792  AJ238793 |
|  | Finnish  (FIN) | AF367064  AF367065  AF367066  AF367068  AF367069  AF367070  AF367071  AJ238788  AJ238789  AJ238790  AJ314597  HE801633  JQ319171  JQ319163  JQ319166  JQ319167  JQ319168  JQ319161  JQ319164  JQ319170  JQ319162  JN831950  Z30702  Z30704  Z46942  Z69985 |
|  | Fusong | EF442087  EF442091 |
|  | Hokkaido | AB010730  AB010731  AB675465 |
|  | Latvian  (LAT) | JN657228  KX757839  KX757840 |
|  | Muju | DQ138128  DQ138133  DQ138140  DQ138142  JX028273  JX046484  JX046487 |

Supplementary Table S2 (continued)

| Hantavirus | Clade/strain | Accession number |
| --- | --- | --- |
| Puumala orthohantavirus | Northern-Scandinavian  (N-SCA) | AJ223371  AM746297  AM746298  AM746311  AM746315  AM746316  AM746317  AM746318  AM746319  AM746320  AM746321  AM746325  AM746329  AM746330  AM746331  AM746332  AM746333  AY526219  GQ339473  GQ339474  GQ339476  GQ339477  GQ339478  GQ339479  GQ339480  GQ339481  GQ339482  J223380  U14137  Z48586 |
|  | Russian (RUS) | AB433845  AJ314598  AJ314599  AF442613  JN657230  JN657231  L08804  M32750  Z21497  Z30706  Z30707  Z30708  Z84204 |

Supplementary Table S2 (continued)

| Hantavirus | Clade/strain | Accession number |
| --- | --- | --- |
| Puumala orthohantavirus | Southern-Scandinavian  (S-SCA) | GQ339483  GQ339484  GQ339485  GQ339486  GQ339487  AJ223368  AJ223369  AJ223376 |
| Tula orthohantavirus | Outgroup sequences | AF017659  AF063892  AF164093  AJ223600  AM945877  Y13979  Z69991 |

**Literature**

1. Saxenhofer M, Schmidt S, Ulrich RG, Heckel G (2019) Secondary contact between diverged host lineages entails ecological speciation in a European hantavirus. PLoS Biology 17 (2):e3000142. doi:10.1371/journal.pbio.3000142

2. Castel G, Couteaudier M, Sauvage F, Pons JB, Murri S, Plyusnina A, Pontier D, Cosson JF, Plyusnin A, Marianneau P, Tordo N (2015) Complete Genome and Phylogeny of Puumala Hantavirus Isolates Circulating in France. Viruses 7 (10):5476-5488. doi:10.3390/v7102884
